# Glycan distribution and density in native skin's stratum corneum

**DOI:** 10.1111/srt.12453

**Published:** 2018-02-07

**Authors:** J. Danzberger, M. Donovan, C. Rankl, R. Zhu, S. Vicic, C. Baltenneck, R. Enea, P. Hinterdorfer, G. S. Luengo

**Affiliations:** ^1^ Center for Advanced Bioanalysis GmbH Linz Austria; ^2^ L'Oréal Research and Innovation Aulnay sous Bois France; ^3^ RECENDT‐Research Center for Non‐Destructive Testing GmbH Linz Austria; ^4^ Institute for Biophysics Johannes Kepler University Linz Linz Austria

**Keywords:** antibody, atomic force microscopy, heparan sulfate, molecular recognition, stratum corneum, topography and recognition imaging, wheat germ agglutinin

## Abstract

**Background:**

The glycosylation of proteins on the surface of corneocytes is believed to play an important role in cellular adhesion in the stratum corneum (SC) of human skin. Mapping with accuracy the localization of glycans on the surface of corneocytes through traditional methods of immunohistochemistry and electron microscopy remains a challenging task as both approaches lack enough resolution or need to be performed in high vacuum conditions.

**Materials and methods:**

We used an advanced mode of atomic force microscope (AFM), with simultaneous topography and recognition imaging to investigate the distribution of glycans on native (no chemical preparation) stripped samples of human SC. The AFM cantilever tips were functionalized with anti‐heparan sulfate antibody and the lectin wheat germ agglutinin (WGA) which binds specifically to N‐acetyl glucosamine and sialic acid.

**Results:**

From the recognition imaging, we observed the presence of the sulfated glycosaminoglycan, heparan sulfate, and the glycans recognized by WGA on the surface of SC corneocytes in their native state. These glycans were found associated with bead‐like domains which represent corneodesmosomes in the SC layers. Glycan density was calculated to be ~1200 molecules/μm^2^ in lower layers of SC compared to an important decrease, (~106 molecules/μm^2^) closer to the surface due probably to corneodesmosome degradation.

**Conclusion:**

Glycan spatial distribution and degradation is first observed on the surface of SC in native conditions and at high resolution. The method used can be extended to precisely localize the presence of other macromolecules on the surface of skin or other tissues where the maintenance of its native state is required.

## 1 INTRODUCTION

The glycosylation of cell membrane proteins plays an important role in cell‐cell interactions, cell adhesion, proliferation, differentiation, morphogenesis, remodeling of the extracellular matrix, hydration, antimicrobial activity,^1^, ^2^ and modulation of inflammatory responses.^3^ In human skin, N‐linked glycosylation is the most common type of post translational modification on Asn‐X‐Ser/Thr motives of epidermal proteins. Here N‐glycans contain a common core pentasaccharide known as the trimannosyl core which can be fucosylated on the first N‐acetyl glucosamine (core fuscosylation). N‐linked glycans are branched oligosaccharides consisting of 3 types: High mannose/oligomannose—consisting, as the name suggests, only of branched mannose chains; Hybrid—addition of N‐acetyl glucosamine, galactose, or fucose to the trimannosyl core or oligomannose structures; Complex—the addition of terminal sialic acids to hybrid N‐glycan structures.^4^

**Figure 1:**
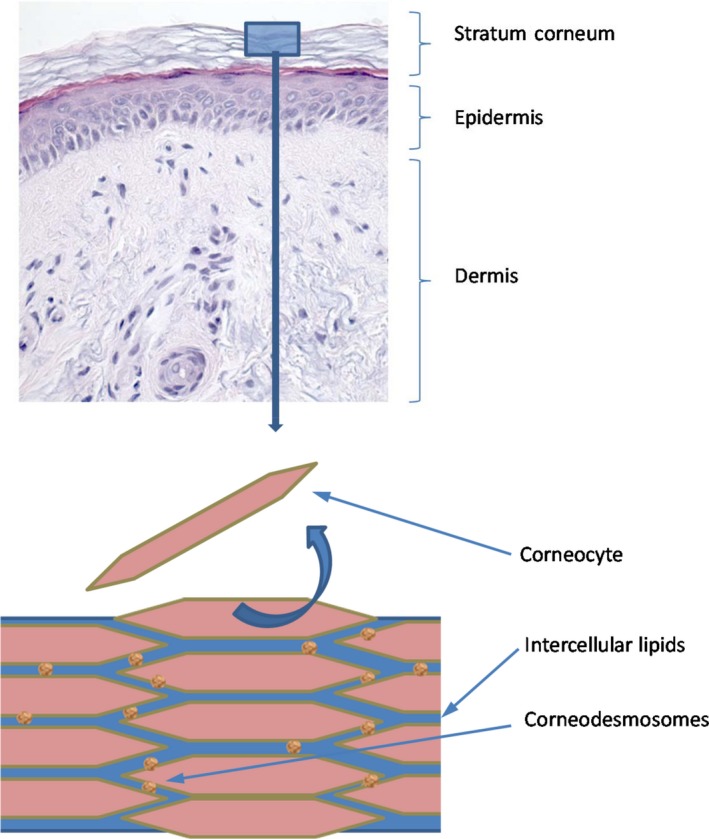
A histological section showing the different compartments of skin—dermis, epidermis, and the outer cornified layer of epidermis—the stratum corneum. Graphic of the stratum corneum showing the organization of corneocytes and lipid layers. Each corneocyte is tethered to a neighboring corneocyte via protein complexes called corneodesmosomes [Colour figure can be viewed at wileyonlinelibrary.com]

A second major glycosylation found on the surface of keratinocytes is the covalent O‐linkage of the sulfated glycosaminoglycan heparan sulfate to Ser/Thr residues of proteins. Heparan sulfated proteoglycans (HSPGs) and N‐linked glycosylated proteins have been postulated to play a role in proliferation and differentiation of the epidermis.^5^, ^6^ In the stratum corneum of human skin (Figure 1), protein glycosylation has been postulated to play an important role in the desquamation process, the shedding of corneocytes from the surface of the skin, since glycan moieties have been demonstrated to protect the corneodesmosomes from proteolysis.^7^ There has been a paucity of information regarding the presence of heparan sulfate or N‐linked glycosylation in the stratum corneum. A histological study of skin sections using plant lectins demonstrated the presence of glyco‐conjugates in the stratum corneum but at a significantly lower level as compared to that in epidermis.^8^ A glycomic study confirmed the presence and changes of N‐linked glycans in the stratum corneum in the dry skin condition, ichthyosis.^9^ A more recent electron microscopic study using plant lectins to detect N‐glycans and an antibody to heparan sulfate evidenced the distribution of these glycans on the surface of delipidated corneocytes.^10^ In these studies, the localization of glycans on the surface of corneocytes through the traditional methods of immunohistochemistry and electron microscopy has required the removal of the lipid‐rich layers of the stratum corneum and may not present a true distribution of these glycans on the SC surface. Thus, in order to obtain a more realistic picture of glycan localization on the surface of native corneocytes, we used an advanced AFM TREC^11^, ^12^ mode with AFM cantilever tips functionalized with anti‐heparan sulfate antibody and WGA, respectively to detect the presence of heparan sulfate and complex N‐glycan chains on the SC surface. This method was proved previously to be well adapted for the study of this skin surface layer.^13^

## 2 RESULTS AND DISCUSSION

### 2.1 Force measurements

**Figure 2:**
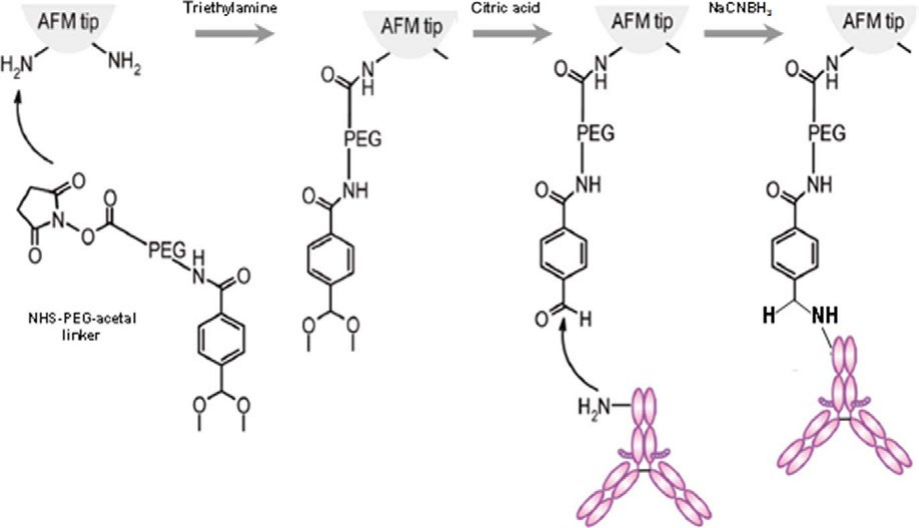
Schematic illustration of the functionalization of the cantilever tip with antibody or WGA. The AFM cantilever tip is first treated with 3‐aminopropyl‐triethoxysilane (APTES) to obtain the amine groups (see Methods) which can react with the NHS group of the PEG linker in the presence of triethylamine. After application of citric acid, the acetal group of the linker converts to aldehyde that subsequently reacts with the amine group of the lysine residue of the anti‐heparan sulfate antibody or WGA. Since both antibody and WGA are coupled to the tip through flexible linkers, they have the possibility to adjust to their ideal orientation for binding to the glycan. In the presence of sodium cyanoborohydride (NaCNBH_3_), the proteins are irreversibly linked to the AFM tip. Their density is sufficiently low to allow for single molecule studies [Colour figure can be viewed at wileyonlinelibrary.com]

**Figure 3:**
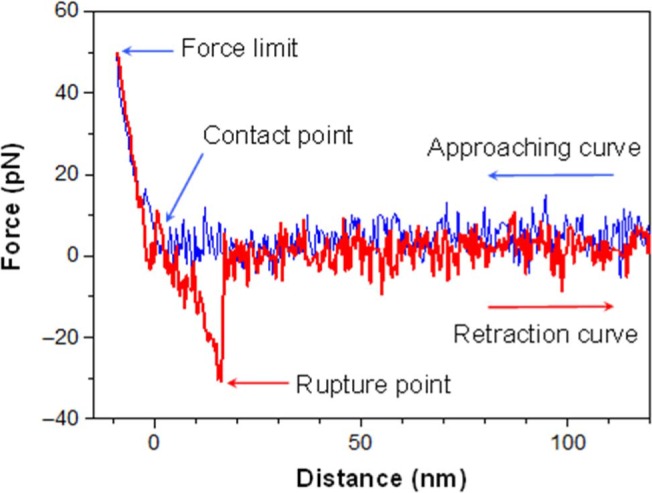
When approaching the cantilever tip, (blue curve), the distance between the tip and the cell surface decreases. At the contact point, the tip touches the cell membrane. After the contact (left side of the contact point), the tip gently presses on the cell membrane. When the force limit (which is about 50 pN here) is reached, the tip is retracted from the cell surface. If the antibody binds to the heparan sulfate, the cantilever tip is pulled downwards (red curve) until the 2 molecules are separated at a critical force. After the rupture, the cantilever recovers its resting state. If there is no binding between antibody and heparan sulfate during the contact, the retraction curve looks similar to the approaching curve [Colour figure can be viewed at wileyonlinelibrary.com]

**Figure 4:**
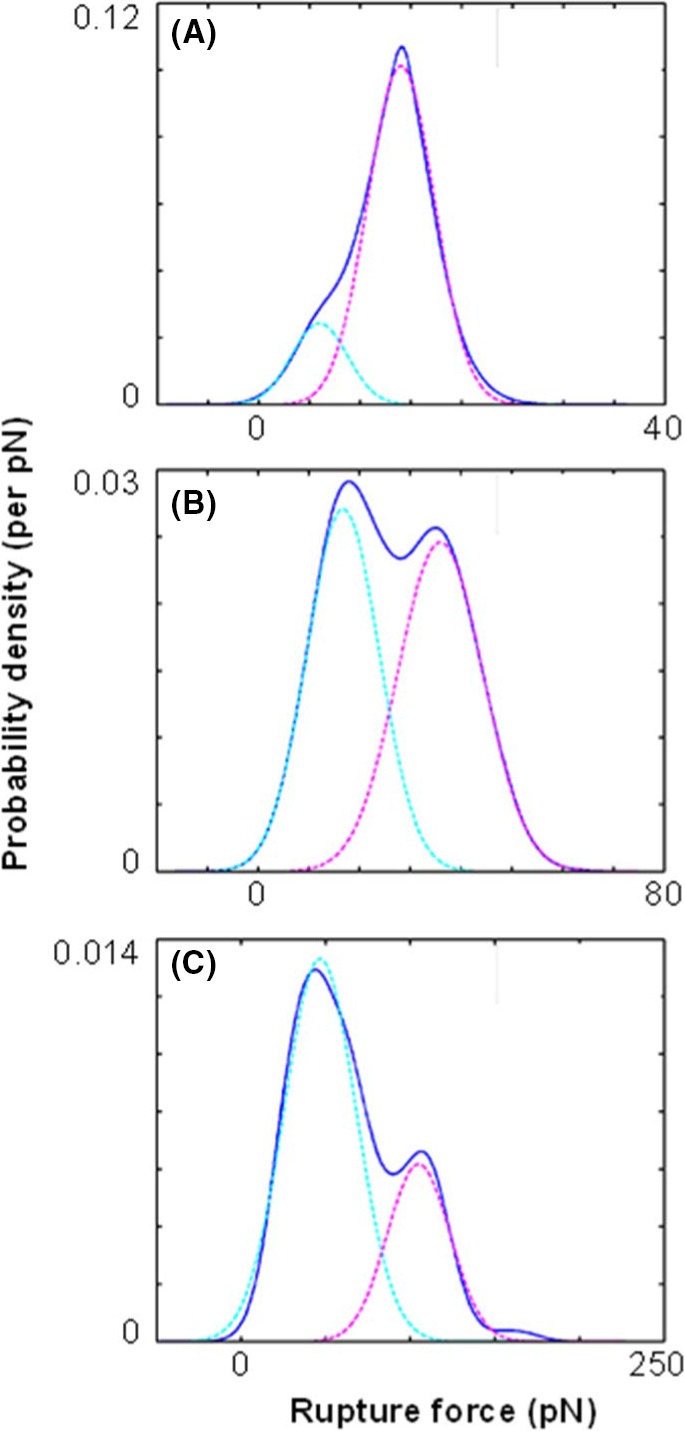
Distribution of rupture forces between a cantilever tip functionalized with monoclonal anti‐heparan sulfate antibody and a stripped abdominal skin sample at different loading rates. The experimentally acquired probability density function (pdf) curves (solid lines) at loading rates of (A) 14, (B) 200, and (C) 16000 pN/s are shown. The measured distributions were fitted with a sum of 2 Gaussians. These Gaussians (dashed lines) are interpreted as single antibody arm unbinding (cyan) and double antibody arm rupture (magenta). The shift of the most probable rupture force for both unbinding modes with increasing loading rates can be seen [Colour figure can be viewed at wileyonlinelibrary.com]

**Figure 5:**
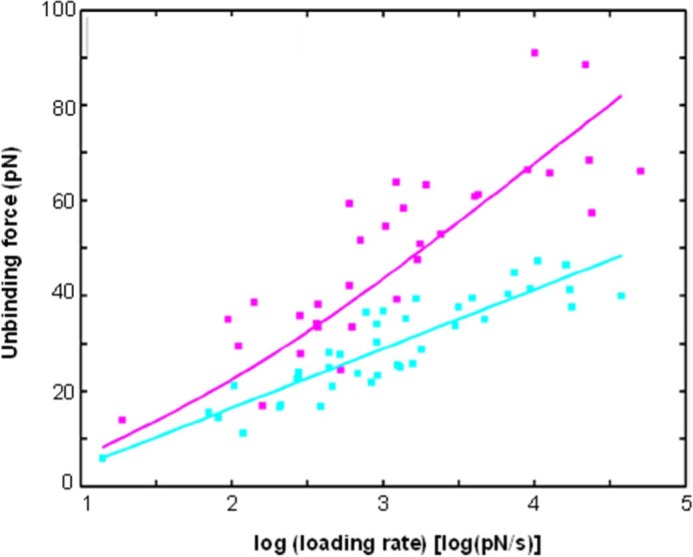
Dynamic force spectroscopy of the anti‐heparan sulfate antibody and its cognate cell receptor. The data for the single arm rupture (cyan squares) were fitted with the single energy barrier model (cyan line). Data of double‐arm ruptures (magenta squares) were fitted using a 2‐bond Markov model (magenta line) with the acquired x_B_ and k_off_ parameters from the single arm rupture fit [Colour figure can be viewed at wileyonlinelibrary.com]

The presence of heparan sulfate on the surface on corneocytes was investigated on stripped abdominal SC samples using single molecular recognition force spectroscopy,^14^ for which the cantilever tips were functionalized with an anti‐heparan sulfate antibody (Figure 2). In this method, the interaction force between the tip and the surface of the corneocyte is measured. An example of force distance curve is shown in Figure 3. The observed binding probabilities were around 15%. The specificity was checked by injecting a solution with free anti‐heparan sulfate antibodies resulting in more than threefold drop of the binding probability. From the force distance curve measurements, mostly single rupture events were observed with the anti‐heparan sulfate antibody. Occasionally, a multistep rupture was obtained. Such events were interpreted as serial unbinding of the individual antibody arms. The distributions of the rupture forces showed a bimodal behavior (Figure 4), which were fitted with a sum of 2 Gaussians. The Gaussian located at lower forces was attributed to rupture events of a single antibody arm. Accordingly, the Gaussian located at higher forces was interpreted as a simultaneous rupture of both antibody arms. The most probable rupture force of the single antibody rupture showed a linear rise with respect to the logarithm of the loading rate. These data were fitted using Evan's single energy barrier model^15^, ^16^

(1) $$F^{*}=\left(k_{B}T/x_{\beta }\right)\ln\left(rx_{\beta }/k_{\mathrm{off}}k_{B}T\right)$$

where F* denotes the most probable rupture force, *r* the loading rate, *k*_off_ the thermal off rate, *k*_B_ the Boltzmann constant, *T* the temperature, and *x*_β_ the thermally averaged length of interaction along the force direction. The fitting parameters *x*_β_ and *k*_off_ were determined to be 0.8 nm and 0.9 s^−1^. The full antibody rupture data were fitted with the uncorrelated Markov model using the above acquired parameters. This represents a configuration for multiple‐bond attachments, where load is shared between all bonds (parallel attachment). Rupture dynamics of multiple bonds depends on the failure mode. In the correlated mode, all bonds are closely coupled, and failure of 1 bond implies failure of remaining bonds. In the uncorrelated system, the attachments can fail independently and the load force is redistributed among surviving bonds. Since both arms of the antibody do not cause close coupling, the analysis for uncorrelated failure was used. Uncorrelated failure mode implies no particular mechanical coupling between individual bonds; it can therefore be described as a Markovian sequence.^17^ The measured unbinding force, ie, most probable rupture force F* scales with the number of bonds, NB and the measured loading rate, *r* according to:

(2) $$r=k_{\mathrm{off}}\frac{k_{B}T}{x_{\beta }}\left[\sum_{l=1}^{N_{B}}\frac{1}{l^{2}}\text{exp}\left(-\frac{F^{*}x_{\beta }}{lk_{B}T}\right)\right]$$

where *x*_β_ and *k*_off_ denote the corresponding parameters derived from a single bond analysis using Equation (1). Figure 5 shows this analysis applied to the full force spectroscopy experiment of single antibody arm and full antibody dissociation. The single arm interaction was fitted using Equation (1), yielding *x*_β_ and *k*_off_. These parameters were used together with Equation (2) to calculate the dynamic force spectrum for full antibody interaction. Both calculation models adequately agree with the obtained data. Our force spectroscopy data provide the proof of principle for the detection of molecular recognition, in which the antibody bound specifically to the heparan sulfate with high affinity. Thus, the functionalized AFM tip is an appropriate tool for localizing recognition sites. The finding of double‐arm binding of the antibody indicates a high surface density of heparan sulfates.

### 2.2 Localization of heparan sulfate and WGA‐binding glycans on corneocytes

Force spectroscopy allowed specific glycans to being detected and identified on the corneocyte surface, yet information about the exact position of the glycans or relation of the glycans with the topography is missing. Combining the topography information of imaging with the identification capability of force spectroscopy allows for specific localization of glycans with corresponding surface features. This is made possible by the simultaneous topography and recognition imaging technique.^11^, ^13^

**Figure 6:**
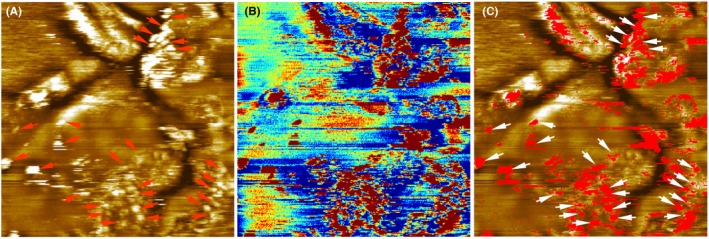
Heparan sulfate mapping of the stripped abdominal skin sample using a cantilever tip functionalized with the anti‐heparan sulfate antibody. The scan size is 1 μm × 1 μm. (A) Topography image. The height scale is 12 nm for the full color range. (B) Simultaneously acquired recognition map. Blue to orange reflects no recognition and dark red corresponds to a recognition event. (C) An overlay of the topography and the recognition map showed that heparan sulfate is located on surface protrusions (bead‐like structures, indicated with arrows) [Colour figure can be viewed at wileyonlinelibrary.com]

At first, a stripped abdominal SC sample was investigated to localize the heparan sulfate on the surface. For this, antibodies against heparan sulfate were tethered to AFM tips as described in the Methods section. The tip used had a spring constant of 0.1 N/m, which was 3‐10 times higher than that of tips used for force spectroscopy. The resulting resonance frequency is about 7.5 kHz in solution and the *Q*‐factor is about 1. The oscillation amplitude was adjusted to be less than the extended PEG linker to provide a proper recognition image.^18^ As a result, the recognition map represents an amplitude reduction due to a physical connection between the heparan sulfate antibody on the tip and heparan sulfate molecules on the skin sample surface when specific interaction occurs. These recognition spots are non‐uniformly distributed as microdomains with dimensions from several nanometers up to 100 nanometers (Figure 6C, red spots). The recognition events correspond with beads observed in the topography image. Repeated scans of the same area showed that the recognition was stable. A threshold analysis was used to identify recognition events. The threshold was set to be the mean value minus 1.414 × SD of this image. Taking into account the size of the tip‐bound molecule (~5 nm) and the length of the PEG linker (~3 nm end‐to‐end distance in solution) allowed the size of a single recognition event to being estimated as a circle of 8 nm radius. Dividing the ratio of the total area of unbinding events to the total scanning area by the area of single molecule recognition spot allowed for the estimation of the glycan density, which was about 1200 heparan sulfate molecules per μm^2^. An overlay of these recognition events with topography showed that almost all recognition sites are on top of topographical elevations. The correlation coefficient between the topography image and the recognition map was determined to be 0.24. This value is significantly different from zero and suggests that recognition events were often found on top of elevated topographical structures, but not all of these structures contain recognition events.

**Figure 7:**
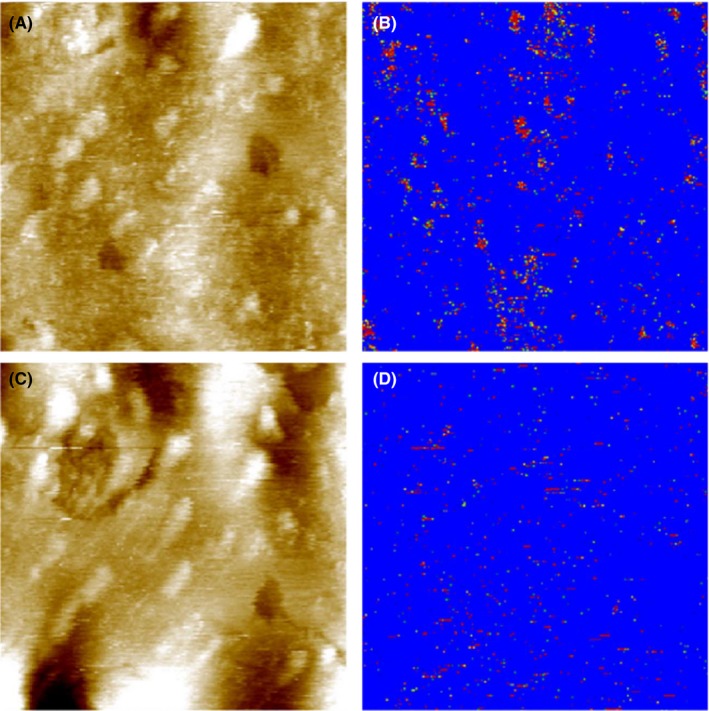
Topography (A and C) and recognition (B and D) images of the second stripped SC layer from the leg of a young volunteer measured with a cantilever tip functionalized with anti‐heparan sulfate antibody. Image size is 0.7 μm × 0.7 μm. The height scale in the topography is 23 nm for the full color range. Before block (A and B), recognition (red‐colored) spots can be seen in the recognition image (B) in the regions corresponding to the protruding bead‐like structures in topography (A). After the injection of 100 μL 1 mg/mL heparan sulfate into the 500 μL measurement solution, the recognition spots were significantly blocked (D) [Colour figure can be viewed at wileyonlinelibrary.com]

**Figure 8:**
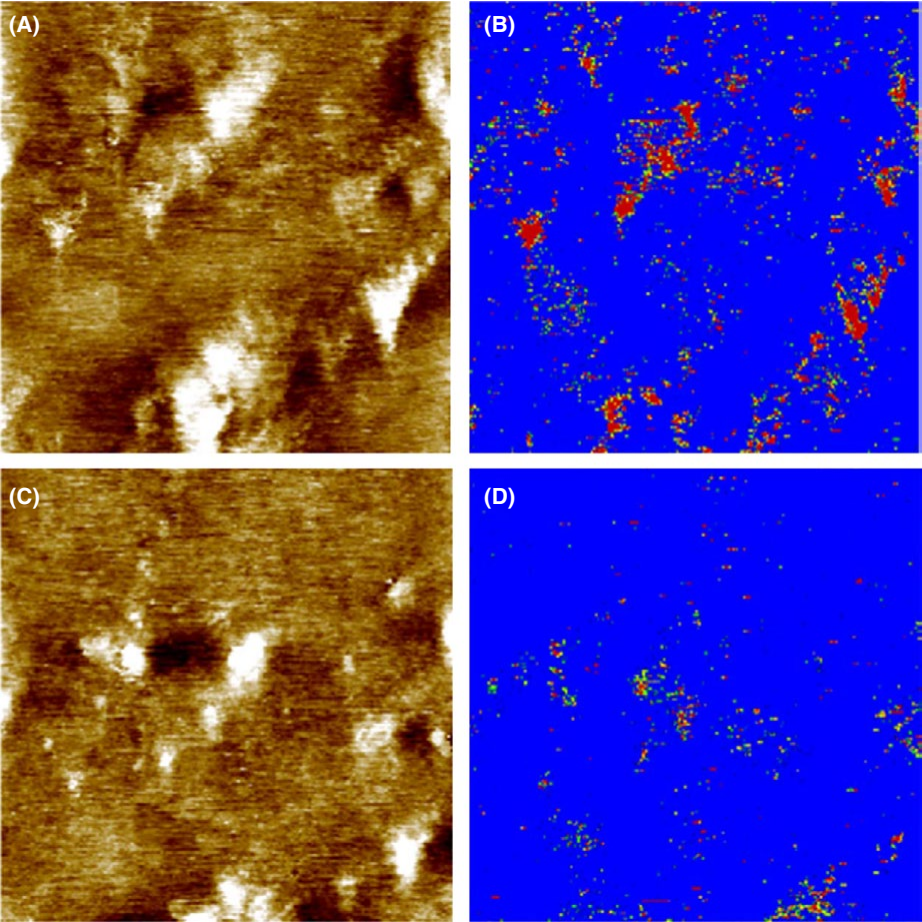
Topography (A and C) and recognition (B and D) images of the second stripped SC layer from the leg of a young volunteer measured with a cantilever tip functionalized with WGA. Image size is 0.9 μm × 0.9 μm. The height scale in the topography is 8 nm for the full color range. Before block (A and B), recognition (red‐colored) spots can be seen in the recognition image (B) in some regions corresponding to the bead‐like protruding structures in topography (A). After the injection of 40 μL 0.7 mg/mL WGA into the 500 μL measurement solution, the recognition spots were significantly blocked (D) [Colour figure can be viewed at wileyonlinelibrary.com]

After the localization of heparan sulfate on the surface of corneocytes on the abdominal SC samples, we extended the investigation to SC skin samples stripped from legs. Figure 7 showed the simultaneously acquired topography and recognition images of the second stripped layer of SC sample from leg measured using a cantilever tip functionalized with the anti‐heparan sulfate antibody. Some of the bead‐like protruding structures in the topography showed recognition spots in the recognition image (in red in the Figure), which indicated that there were binding events between the functionalized anti‐heparan sulfate cantilever tip and the corneocyte surface during the scanning. Notably, not all bead‐like protruding membrane regions contained recognition spots. To examine the specificity of the interaction, the block experiment was performed by in situ injection of 100 μL free heparan sulfate (1 mg/mL) into the 500 μL measurement solution. After the injection, the bead‐like protruding membrane profile in the topography image remained the same. However, the number (or density) of recognition spots were significantly reduced in the recognition image. The successful blocking experiment indicated that the detected recognition spots were due to the specific interaction between heparan sulfate on the corneocyte surface and the anti‐heparan sulfate antibody on the cantilever tip. In Figure 8, the second layer of the SC sample from the leg was measured by a cantilever tip functionalized with WGA. Some of the bead‐like protruding regions showed recognition (red) spots in the recognition image. After the in situ injection of 40 μL free WGA (0.7 mg/mL) into the measurement solution, the number of recognition spots was significantly reduced.

From all these TREC measurements, we can conclude that glycans on SC layers were associated with the elevated bead‐like features on the surface. Both heparan sulfate and the WGA‐binding glycans were found on the surface of corneocytes of SC layers. They were localized exclusively in some bead‐like protruding regions of the membrane that are similar to those in Rankl et al,^13^ which were identified to be corneodesmosome by using specific antibody. Notably, not all of the corneodesmosome‐like regions were covered with glycans.

In the present study, the atomic force microscope was used to investigate the presence of glycans on the surface of corneocytes in different SC samples. AFM's imaging capabilities allow for a precise mapping of the morphology of the corneocyte surface in the SC. One prominent surface feature was observed, ie, the clusters of protruding bead‐like structures at the surface of the SC. The height of these bead‐like structures varied from 1.1 to 4 nm. These bead‐like structures are similar to the structures described on SC corneocytes in a previous study^13^ and may correspond to remnants of corneodesmosomes or similar adhesive structures on the corneocyte surface. This is supported by the fact that in electron microscopy experiments, similar bead‐like structures were found and attributed to intercellular junctions.^19^ Corneodesmosomes are progressively degraded during the desquamation process^20^, ^21^ and thus are ultimately smaller in size and sparser in density at the upper layers of the stratum corneum compared to lower ones. Hence, in AFM the scattered small bead‐like structures observed in the stripped SC samples may correspond to partially degraded corneodesmosomes. Specific cell membrane molecules have been localized using optical methods such as immunofluorescence staining or electron microscopy. However, these techniques lack information about topography of investigated molecules on the surface of a cell, and the lateral resolution of conventional fluorescence microscopy is not better than 200 nm. At present, AFM offers a unique solution to obtain topographical images with nanoscale resolution without requiring rigorous sample preparation or labeling. The recently developed technique of simultaneous TREC allows quick and easy mapping of binding sites to being achieved with a lateral accuracy of several nanometers across a variety of surfaces.^13^, ^22^, ^23^ Examination of the topography and recognition images of stripped SC layers showed that the glycan moieties were exclusively localized on the bead‐like protruding regions which were possibly corneodesmosomes. But not all bead‐like structures showed recognition. About 106 heparan sulfate molecules per μm^2^ (Figure 7) and 111 WGA recognized glycan molecules per μm^2^ (Figure 8) were found for the second stripped SC samples from the leg. Compared to the 5th‐6th stripped SC sample from abdominal skin (Figure 6), the density of glycans in the upper layer decreased dramatically. Corneodesmosomes are major components of intercellular junctions between corneocytes in SC. Some of the key components of corneodesmosomes desmoglein 1, desmocolin 1, and corneodesmosin are N‐linked glycosylatyed proteins.^24^, ^25^, ^26^ Thus, during the desquamation process, corneodesmosomes are gradually degraded by a sequential action of glycosidases and proteases. Both the size and the density of corneodesmosomes decrease from the inner SC layer to the outer SC layer (Figure S1 and 9).

**Figure 9:**
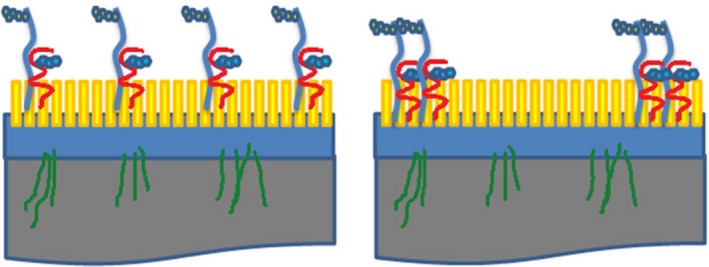
A diagram showing the distribution of glycans on the surface of corneocytes from the lower (left panel) and upper (right panel) layers of the stratum corneum. Heparan sulfate proteoglycans (shown in blue) and the glycosylated corneodesmosin proteins such as corneodesmosin (shown in red) are distributed evenly on the surface of corneocytes in the lower layers of the stratum corneum, whereas in the upper layers, these glycans are found typically at the periphery of corneocytes [Colour figure can be viewed at wileyonlinelibrary.com]

Our results confirm the presence of heparan sulfate and glycans which can be recognized by WGA, on the surface of SC corneocytes. In previous studies,^8^, ^10^ the localization of glycans on the surface of corneocytes was shown through the traditional methods of immunohistochemistry and electron microscopy, which required the removal of the lipid‐rich layers of the stratum corneum and may not show a true distribution of these glycans on the SC surface. Here we mapped glycans on the surface of SC corneocytes in their native state. Moreover, using TREC we correlated the recognized glycan sites with the bead‐like structure of corneodesmosomes in the SC.

It has been shown that both peripheral and non‐peripheral corneodesmosomes persist at the corneocyte surface in the upper SC layers of xerotic skin.^19^ Thus, AFM is a very useful tool to characterize the morphological changes and the distribution of other corneodesmosomal components (eg, desmoglein and desmocolin) and various glycan moieties (eg, hyaluronan) on the surface of corneocytes. Such information will aid designing new skincare—compounds prone to better interact with the SC surface.

## 3 METHODS

### 3.1 Sample preparation

For TREC imaging, a varnish stripping method was used to collect SC samples from the surface of the skin from legs of 6 young volunteers (20‐25 years) and 6‐old volunteers (60‐65 years). A nylon membrane (50 cm^2^) was placed on the surface of the skin and varnish was applied by a brush. After 10 minutes, the membrane was peeled off. Samples were transported in dry ice and stored at −80°C. Prior to AFM measurements, they were thawed to room temperature in a box with silica dry gel. For force measurements, SC samples from abdominal plastic surgery were used. SC sheets were cut into small pieces and set on a thermonox support using a fine layer of cyanoacrylate glue. Stripping was carried out by mechanical removal of the first 5‐6 SC layers using a scotch tape.

### 3.2 Tip functionalization

For TREC imaging, the anti‐heparan sulfate antibody and the plant lectin wheat germ agglutinin (WGA) were covalently bound (as illustrated in Figure 2) to commercially available type VII Maclevers (Agilent Technologies Inc., Chandler, AZ, USA) via a hetero‐bifunctional poly(ethylene glycol) (PEG) crosslinker.^27^ Briefly, the cantilevers were washed 3 times in chloroform, treated with ozone plasmatron for 15 minutes, and further washed with chloroform for 3 times. Then the cantilevers were treated with 3‐aminopropyl‐triethoxysilane (APTES) in gas phase^28^ and washed once with chloroform. NHS‐PEG29‐acetal crosslinker (1 mg) was dissolved in 0.5 mL chloroform before 10 μL triethylamine was added. The cantilevers with APTES coating were incubated in the crosslinker solution for 2 hours. Afterwards, they were washed 3 times in chloroform and dried in air. The cantilevers were then treated with 1% citric acid for 10 minutes, washed threefold in Millipore water and once in ethanol, then dried in air. For functionalization with WGA, the cantilevers were incubated in 20 μL of 0.7 mg/mL WGA solution on a piece of clean parafilm in a 35 mm plastic Petri dish. 1 μL 1M sodium cyanoborohydride (NaCNBH_3_, 32 mg) dissolved in the mixture of 50 μL 100 mM NaOH and 450 μL Millipore water was added to the WGA solution. During the 1‐hour incubation, the dish was sealed with parafilm. Afterwards, the cantilevers were washed threefold in 3 mL phosphate‐buffered saline (PBS), and stored in PBS at 4°C before measurement. For recognition imaging on samples from leg, cantilevers were also functionalized with antibody against heparan sulfate. About 10 μL 1 mg/mL Heparan sulphate monoclonal antibody (OBT1698, AbDserotec) was dialyzed in 250 mL PBS (PAA) 2 times to remove sodium azide. The first dialysis took 2 hours and the second about 19 hours. After the dialysis, the volume of the antibody solution increased to 110 μL. About 4 μL 1M NaCNBH_3_ solution was added into the antibody solution and the cantilevers with crosslinker were incubated in the antibody solution for about 4 hours. Subsequently, the cantilevers were washed in PBS for 3 times, and stored in PBS at 4°C before measurement. The used NaCNBH_3_ solution was treated with sodium bicarbonate (Na_2_CO_3_) solution (3 g solved in 1 L water) with stirring for 1 hour to remove toxicity. The cantilever tips for force measurements were prepared by functionalization with monoclonal anti‐heparan sulfate antibody (AbD Serotec, T320.11) on commercially available cantilevers (MSCT, Veeco Instruments Inc., Santa Barbara, CA, USA) using the similar procedure. The cantilever tips for recognition imaging on abdominal samples were prepared by functionalization with monoclonal anti‐heparan sulfate IgM (provided by L'Oréal Laboratories) on type VII Maclevers (Agilent Technologies Inc.) using the similar procedure.

### 3.3 AFM experiments

All TREC measurements were taken using a PicoPlus 5500 AFM (Agilent Technologies Inc. mentioned above). Topography and recognition images were measured in magnetic AC (MAC) mode making use of the commercially available PicoTREC module (Keysight Technologies Inc., Chandler, AZ, USA). TREC images were recorded using magnetically coated cantilevers with 0.1‐0.14 N⁄m nominal spring constant, at an oscillation frequency of 7.5‐8.9 kHz. The oscillation amplitude of the cantilever was adjusted to provide proper recognition images.^18^ The setpoint of the imaging feedback loop was set as close as possible to the free oscillation amplitude to minimize crosstalk between topography and recognition. Scanning speed was typically 1 μm/s. All TREC images were acquired in PBS. The oscillation signal of the cantilever was split into the upper and lower parts in the PicoTREC module. The upper part of the oscillation signal was used to construct the recognition image. When the tethered molecule on the cantilever tip bound with the sugar moiety on the cell membrane, the upper part of the cantilever oscillation was reduced, resulting in a dark spot in the recognition image. For the blocking experiment, 100 μL 1 mg/mL heparan sulfate (H7640, Sigma, Saint Louis, MO, USA) or 40 μL 0.7 mg/mL WGA was injected into the ~500 μL measurement solution. For contact mode imaging in air, MSCT cantilever (Bruker, Camarillo, CA, USA) with a nominal spring constant of 0.03 N/m was used. The force setpoint was adjusted to very low forces. Force distance cycles were acquired by approaching the antibody modified tip to a stripped skin sample immersed in PBS and retracting it. For force spectroscopy experiments, force distance cycles with a sweep range of 400 nm and sweep rates of 0.1‐10 Hz were performed at room temperature using antibodies coupled with AFM tips with 0.01‐0.03 N⁄m nominal spring constants. The cantilever spring constants were determined using the thermal noise method.^29^, ^30^ Empirical force distribution of the rupture forces of the last unbinding event (pdf) were calculated as previously described.^31^ The pdfs were fitted with the multiple Gaussian function. The loading rates were determined by multiplying the pulling velocity with the effective spring constant, ie, the mean slope at rupture.
