# Supplementary figures and images for: Glycan distribution and density in native skin's stratum corneum
Source: Skin Res Technol. 2018 Feb 7;24(3):450–8. doi: 10.1111/srt.12453 (PMC6446803; doi:10.1111/srt.12453)

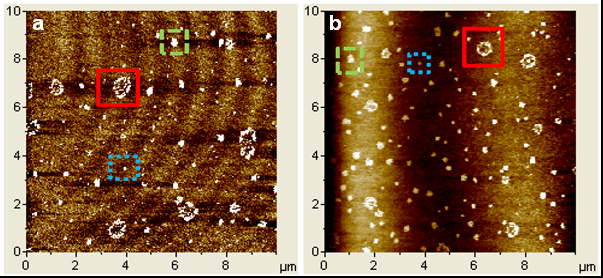

Supplement: Supplementary file 1 [file SRT-24-450-s001.tif]
